# Challenges to dialysis treatment during the COVID-19 pandemic: a qualitative study of patients’ and experts’ perspectives

**DOI:** 10.3389/fpsyg.2023.1185411

**Published:** 2023-11-15

**Authors:** Krystell Oviedo Flores, Tanja Stamm, Seth L. Alper, Valentin Ritschl, Andreas Vychytil

**Affiliations:** ^1^Division of Nephrology and Dialysis, Medical University of Vienna, Vienna, Austria; ^2^Baxter Healthcare GmbH, Vienna, Austria; ^3^Institute of Outcomes Research, Center for Medical Data Science, Medical University of Vienna, Vienna, Austria; ^4^Ludwig Boltzmann Institute for Arthritis and Rehabilitation, Vienna, Austria; ^5^Division of Nephrology, Beth Israel Deaconess Medical Center and Department of Medicine, Harvard Medical School, Boston, MA, United States

**Keywords:** anxiety, COVID-19, dialysis experts, hemodialysis, isolation, peritoneal dialysis, qualitative study

## Abstract

**Background:**

The global COVID-19 pandemic transformed healthcare services in ways that have impacted individual physical and psychological health. The substantial health challenges routinely faced by dialysis-dependent patients with advanced kidney disease have increased considerably during the ongoing COVID-19 pandemic but remain inadequately investigated. We therefore decided to analyze and compare the perspectives of dialysis patients on their own needs and challenges during the COVID-19 pandemic with those of their professional healthcare providers through interviews with both groups.

**Methods:**

Qualitative study of seven in-center hemodialysis patients, seven peritoneal dialysis patients, seven dialysis nurses, and seven physicians at the Medical University of Vienna between March 2020 and February 2021, involving content analysis of semi-structured interviews supported by a natural language processing technique.

**Results:**

Among the main themes emerging from interviews with patients were: (1) concerns about being a ‘high-risk patient’; (2) little fear of COVID-19 as a patient on hemodialysis; (3) questions about home dialysis as a better choice than in-center dialysis. Among the main themes brought up by physicians and nurses were: (1) anxiety, sadness, and loneliness of peritoneal dialysis patients; (2) negative impact of changes in clinical routine on patients’ well-being; (3) telehealth as a new modality of care.

**Conclusion:**

Preventive measures against COVID-19 (e.g., use of facemasks, distancing, isolation), the introduction of telemedicine, and an increase in home dialysis have led to communication barriers and reduced face-to-face and direct physical contact between healthcare providers and patients. Physicians did not perceive the full extent of patients’ psychological burdens. Selection/modification of dialysis modality should include analysis of the patient’s support network and proactive discussion between dialysis patients and their healthcare providers about implications of the ongoing COVID-19 epidemic. Modification of clinical routine care to increase frequency of psychological evaluation should be considered in anticipation of future surges of COVID-19 or currently unforeseen pandemics.

## Introduction

SARS-CoV-2 (World Health Organization, 2020) has transformed healthcare delivery in many countries. The impact of coronavirus disease (COVID-19) strained healthcare infrastructure, resources, and personnel in various ways. Disruption in healthcare, delayed diagnoses, postponed surgical procedures, temporary suspension of transplantation programs, and barriers to accessing healthcare services affected patients with chronic underlying conditions.

Patients with advanced chronic kidney disease (CKD) constitute a particularly vulnerable group. For those who require dialysis as life-sustaining therapy, missing treatment sessions or interruption of treatment could lead to serious health consequences. Many of the patients waitlisted for kidney transplantation must undergo dialysis. Dialysis patients experience multiple comorbidities and are often immunocompromised. They require regular medical appointments for dialysis sessions, laboratory tests, and other healthcare needs. Frequent exposure to healthcare settings could increase the risk of exposure to the SARS-CoV-2 virus. These conditions increase the risk of a COVID-19 infection associated with higher mortality in these patients (Wang et al., 2020; Centers for Disease Control and Prevention (CDC), 2020; Huang et al., 2020).

The choice between dialysis modalities depends on the patient’s overall health, comorbid conditions, lifestyle, preference, and other medical considerations. Peritoneal dialysis (PD) is a kidney replacement therapy that requires instillation of glucose-containing fluids into the peritoneal cavity via an abdominal catheter that comes in contact with the peritoneal membrane. Excess fluid is eliminated by osmotic ultrafiltration (via the osmotic gradient between blood and PD fluid), while uremic toxins are removed by diffusion from the peritoneal wall capillaries (a natural filter) into the peritoneal cavity. PD fluid is manually drained from the peritoneal cavity after several hours and replaced by fresh solution (e.g., four times/day). Alternatively, patients may undergo treatment with a machine (a “cycler”) that automates peritoneal fluid exchanges through the night, bypassing the need for daytime manual fluid exchanges. After training, patients perform PD at home. PD requires regular delivery of supplies such as PD solutions or hand disinfectants at home. As home therapy, PD offers more autonomy and independence to patients and requires follow-ups at the outpatient clinic approximately every six weeks. However, this type of treatment requiring high degrees of responsibility for oneself and adherence to medical protocol is not suited to all patients dependent on dialysis for renal replacement therapy.

Hemodialysis (HD) can also be provided at home. However, in most countries, patients are treated in the hospital or dialysis center on an outpatient basis (in-center HD). HD patients require a vascular access (fistula or central venous catheter). Via this access, blood is removed from the patient’s bloodstream to flow through and be filtered by the hemodialysis machine, for subsequent reinfusion into the patient’s bloodstream. Uremic toxins and excess fluids are removed from the patient’s bloodstream by diffusion and hydrostatic ultrafiltration. In-center HD patients have three times weekly dialysis schedules at the dialysis center. Each session lasts three to five hours. Since in-center HD is delivered by nurses, patients are often passive recipients of treatment in a clinical setting, with less direct involvement in the dialysis process itself. The environment at the dialysis unit involves close contact with healthcare staff and other patients. For patients living alone, in-center HD sometimes provides a social network.

During the pandemic, dialysis patients have been subjected to stricter regulations at healthcare institutions, such that face-to-face contact has been partially replaced by remote monitoring and telemedicine to reduce virus transmission (Weiner and Watnick, 2020; Antoun et al., 2021). Guidelines and recommendations since the COVID-19 pandemic have promoted the transition to home dialysis (either PD or home HD) to reduce patients’ exposure to COVID-19 infection [Brown et al., 2020; Cozzolino and ERA-EDTA Council, 2020; National Institute for Health and Care Excellence (NICE), 2020; The UK Kidney Association, 2020; Cozzolino et al., 2021]. Social distancing measures aimed at reducing spread of the virus resulted in increased social isolation, which could impact the mental and emotional well-being of patients with chronic diseases. The association between chronic kidney disease and clinical severity of COVID-19 infection and the lack of information related to changes in healthcare delivery has imposed on dialysis patients significant burdens of anxiety and fear (Henry and Lippi, 2020; Xia et al., 2021). Increased communication and information from healthcare providers remain essential for optimal patient management.

One previous study has examined patient and clinician perspectives on the transition to dialysis in patients >70 years of age with CKD stages 4 or 5 (Porteny et al., 2022). Other reports have studied kidney transplant candidates (Guha et al., 2020), in-center HD patients (Sousa et al., 2021; Malo et al., 2022), or their caregivers (Sousa et al., 2022). However, no study to date has applied a qualitative analytic approach to compare perspectives of HD and PD patients with those of their healthcare providers during the COVID-19 pandemic. We therefore conducted this qualitative interview study to explore the needs and challenges faced during the COVID-19 pandemic by PD and in-center HD patients. We also solicited the opinions of dialysis nurses and physicians on the challenges faced by their patients.

## Methods

### Study design, setting, and participants

This qualitative study included 28 participants interviewed during the COVID-19 pandemic between March 2020 and February 2021 at the Division of Nephrology and Dialysis, Medical University of Vienna, Austria. We recruited fourteen patients in maintenance dialysis, seven treated with in-center HD and seven treated with PD. Patients were selected using convenience sampling, including a broad spectrum of age, years of dialysis treatment, and comorbidities. Patients with severe cognitive impairment were excluded. Also included were seven nephrologists or nephrology trainees and seven nurses with experience in both dialysis modalities, with a wide range of seniority and experience. Patients were informed about the study and invited to participate by phone or during a routine hospital visit. A single one-on-one interview by appointment at the participant’s choice was conducted at our center. All participants provided written informed consent. The study was performed according to the principles of the Declaration of Helsinki. Approval was granted by the intra-university Data Protection Committee and the Local Ethics Committee of the Medical University of Vienna (study protocol EK 1725/2020).

### Interview guides and data collection

Qualitative content analysis is suitable for our exploratory approach to understanding behaviors associated with a human condition in different contexts and perceived situations (Kvale, 1996; Ritschl and Stamm, 2016). Based on an anonymous questionnaire developed to improve routine care of dialysis patients at the outbreak of the COVID-19 pandemic, we created topic guides that covered aspects related to sources of information, preventive measures against COVID-19, problems with consumables (e.g., hand disinfectants, PD solutions), patients’ experience at the dialysis unit, and psychological aspects (Supplementary Tables S1, S2). An experienced qualitative researcher (T.S.) reviewed and adapted the interview guides. Interviews started with an open question as an ice-breaker to motivate participants to talk (for example: *“What is the most challenging as a patient to deal with during COVID-19?”*). We formulated questions in a neutral form, trying to explore not only negative but also positive aspects related to the COVID-19 pandemic (for example: *“Is there something you liked (or disliked) about the patient healthcare/during the lockdown period?”*). To minimize reporting bias, all interviews were performed by K.O.F (female, MD, MSc, and candidate for the Ph.D. degree), who was uninvolved in the patients’ care and otherwise unrelated to the patients. Semi-structured interviews conducted at the center were audio-recorded and transcribed verbatim. Demographic characteristics were taken from patient clinical records with written consent of the patients. We reported the study following the Consolidated Criteria for Reporting Qualitative Health Research (COREQ) Guidelines (Tong et al., 2007) (Supplementary Table S3).

### Analyses

Descriptive statistics were used to summarize participants’ characteristics using GraphPad Prism Software, version 9.0.1. Results were expressed as relative frequencies for categorical variables, as means with standard deviations (SD) for continuous variables, and as medians with interquartile ranges (IQR) for skewed distributions. Fisher’s exact test was used to compare categorical variables. Continuous variables were analyzed by Mann–Whitney U-test. Two-tailed tests were used for all comparisons. We conducted an inductive thematic analysis of qualitative data to discover topics describing patients’ experiences and perceptions of their dialysis during the COVID-19 pandemic, followed by a modified meaning condensation form (Stamm et al., 2011; Bengtsson, 2016). All transcripts were carefully read and checked for accuracy. Coding was performed independently by K.O.F. and A.V. Data were divided into meaning units (defined as specific text parts, either a few words or sentences with a common meaning) summarized in one or more concepts. Associated concepts were grouped, and a scheme of lower- and higher-level concepts was developed. Lower-level concepts share the attributes of the higher-level concepts but are more specific. An additional researcher (V.R.) with extensive experience in qualitative research reviewed the results. Extensive discussion among V.R., A.V., and K.O.F. resolved disagreements to reach a consensus through the triangulation technique.

We used Atlas.ti version 8 [ATLAS.ti Scientific Software Development GmbH, 2018] for analysis and coding. Additionally, we used a natural language processing technique called Latent Dirichlet Allocation (LDA) to triangulate the thematic analysis and uncover potential additional topics (Gefen et al., 2017; Ruiz et al., 2020). LDA is an unsupervised, generative, probabilistic topic modeling technique that extracts meanings from a pre-defined number of topics/concepts. The number of concepts resulted from the most evident differentiation in the representation of heat maps and the examination of words (topic coherence). As an unsupervised machine learning technique, LDA’s primary advantage is its independence from prior knowledge or predefined categories. For this purpose, we created a semantic space by stemming the words, removing stop words (such as ‘and’, ‘the’, ‘a’, and similar), and converting all text to lowercase. In addition, punctuation marks, names, and personal words were removed from the text. LDA analyzes texts by considering word frequency in combination with the co-occurrence of words. LDA characterizes concepts based on word frequency and the words that best distinguish one concept. We conducted separate analyses for physicians, nurses, HD patients, and PD patients, assuming these four groups had different experiences during the two COVID-19 waves. R software (R Development Core Team, 2008) was used to conduct the LDA.

## Results

The median age of HD patients was 54 years (IQR 43) and that for PD patients was 56 years (IQR 15) (*p* > 0.99). Other baseline data revealed statistically significant differences between groups only for hypertension (PD versus HD patients, *p* = 0.02). The median age of nurses was 51 years (IQR 5), and of physicians, 35 years (IQR 12) (*p* < 0.001). Most healthcare providers (71%) had more than five years of work experience in dialysis units. All patients were Caucasian. Other participant characteristics are shown in Table 1. The average interview length was 66 min (with a range of 46 to 106 min).

**Table 1:** Participants’ characteristics.

Characteristic	HD patients (*n* = 7)	PD patients (*n* = 7)	Nurses (*n* = 7)	Physicians (*n* = 7)
Age in years, median (IQR)	54 (43)	56 (15)	**51 (5)**	**35 (12)**
Sex, *n* (%)				
Female	3 (42.8)	4 (57.2)	5 (71.4)	4 (57.2)
Male	4 (57.2)	3 (42.8)	2 (28.6)	3 (42.8)
Marital status, *n* (%)			N/A	N/A
Married/Partnered	6 (85.7)	7 (100)		
Single	1 (14.3)	0		
Employment status, *n* (%)			N/A	N/A
Employed	2 (28.6)	3 (42.8)		
Not employed	0	1 (14.3)		
Retired	5 (71.4)	3 (42.8)		
Living alone			N/A	N/A
Yes	0	0		
No	7 (100)	7 (100)		
Living conditions, *n* (%)			N/A	N/A
House (with garden)	4 (57.2)	7 (100)		
Flat	2 (28.6)	0		
Shared-flat	1 (14.3)	0		
Time on dialysis in years, median (IQR)	1.25 (5.5)	3.5 (2.2)	N/A	N/A
Years of working experience in dialysis, *n* (%)	N/A	N/A		
Less than 5 years			3 (42.8)	1 (14.3)
From 6 to 10 years			3 (42.8)	0 (0)
More than 10 years			1 (14.3)	6 (85.7)
Cause of chronic kidney disease, *n* (%)			N/A	N/A
Glomerulonephritis	3 (42.8)	2 (28.6)		
Polycystic kidney disease	1 (14.3)	1 (14.3)		
Diabetic nephropathy	0 (0)	1 (14.3)		
Vascular nephropathy	1 (14.3)	0 (0)		
Other/Unknown	2 (28.6)	3 (42.8)		
Comorbidities, *n* (%)			N/A	N/A
Hypertension	**7 (100)**	**2 (28.6)**		
Diabetes	0 (0)	1 (14.3)		
Cancer	1 (14.3)	0 (0)		
Cardiovascular disease	5 (71.4)	3 (42.8)		

HD, (in-center) hemodialysis; PD, peritoneal dialysis.

When comparing values of HD vs. PD patients, and values of nurses vs. physicians, p-value was calculated using the Fisher’s exact test for categorical variables, and the Mann–Whitney U test for continuous variables; significant results are highlighted in bold.

### Key themes raised by HD and PD patients

**Figure 1:**
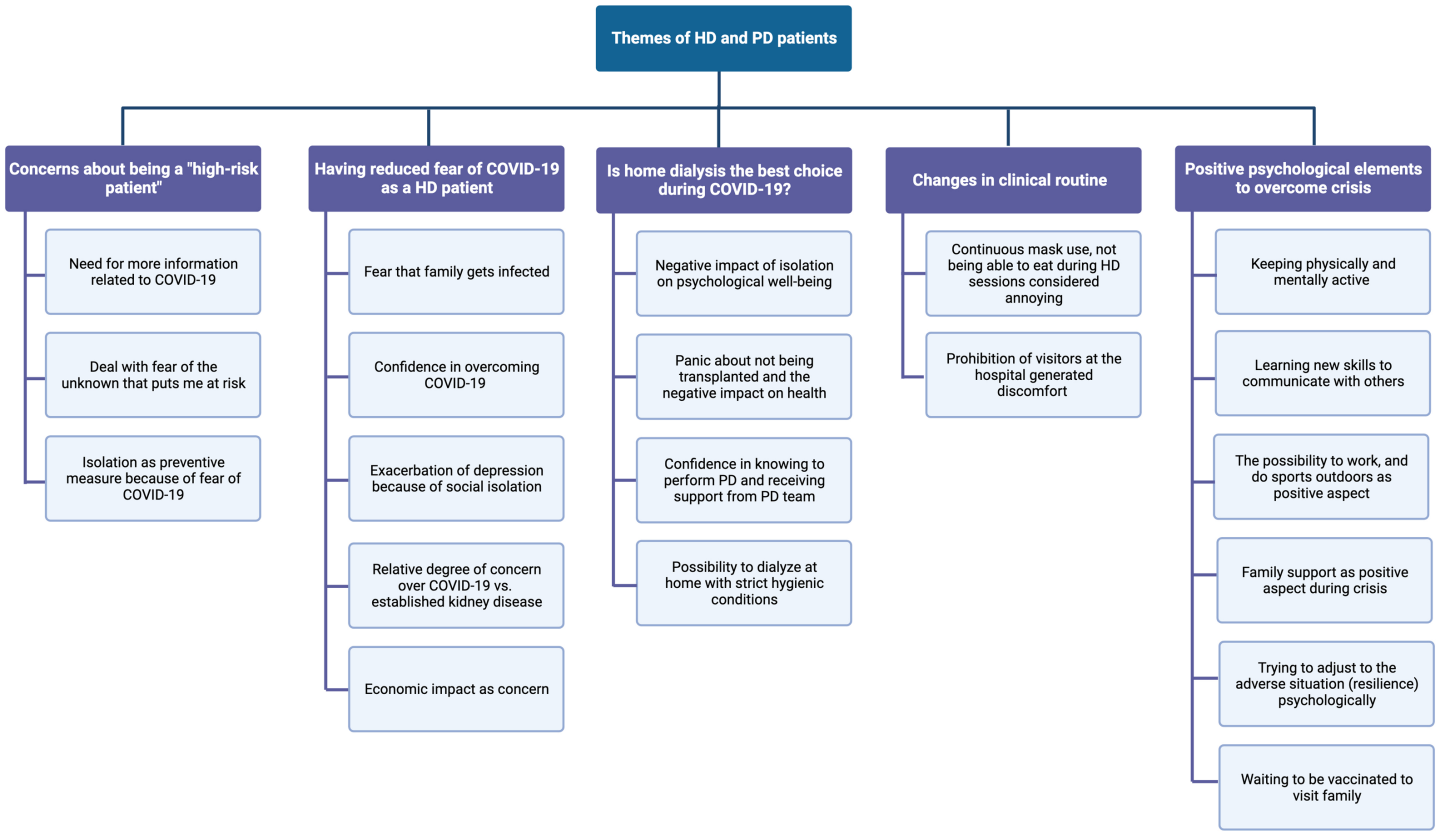
Coding tree identified through content analysis and natural language processing (LDA) of the interviews of dialysis patients.

Five main themes and subthemes characterized PD and HD patients’ experiences: (1) concerns about ‘high-risk patient’ status (lack of information about implications of COVID-19 infection in high-risk patients); (2) little fear of COVID-19 as a patient on HD (more significant concern about personal difficulties related to COVID-19 or kidney disease); (3) questions about home dialysis as a better choice during COVID-19 (home therapy kept patients away from contact with hospital, increasing anxiety; is the autonomy of PD treatment an advantage?); (4) changes in clinical routine (limitations and changes during dialysis routine upset patients and their family); (5) positive psychological elements to overcome the crisis (alternative routines to promote well-being, cultivating positive emotions to transform suffering, seeing the glass half full, vaccination as hopeful for patients at risk). All topics are presented in a thematic coding tree (Figure 1), and exemplary quotes are included in Table 2.

**Table 2:** Main themes with subthemes and exemplary quotes of peritoneal dialysis (PD) and hemodialysis (HD) patients.

Theme complexes	Main themes	Subthemes	Quotation (Participant Role, Sex, Age)
Concerns about Being a ‘High-Risk Patient’	Lack of information related to COVID-19 implications for high-risk patients	Need for more information related to COVID-19 and special measures for high-risk patients	*“During the doctor’s consultation, we did not explicitly discuss possible consequences of the virus for high-risk patients or whether I should comply with special measures. It would be good if someone addressed whether I have to pay special attention to certain things.”* (PD patient, female, age 52)
		Deal with fear of the unknown that puts me at risk	*“Dealing with the fear was the biggest challenge for all of us because a virus massively restricted our lives. Nobody told me to do this and that in my situation to protect myself. I have no idea how bad this virus is and how it endangers me.”* (PD patient, male, age 50)
		Isolation as a preventive measure because of fear of COVID-19	*“I moved into an empty house and lived alone for three months just to be safe. I have a family, a wife, and two children who can bring the virus home. I saw nobody, met nobody, nothing at all. I hardly went out on the street, either.”* (PD patient, male, age 50)
Having Reduced Fear of COVID-19 as a HD Patient	Greater concern about personal difficulties related to COVID-19 or kidney disease	Fear that family gets infected	*“What scared me mostly was not being able to see my kids and friends.”* (HD patient, female, age 69)
		Confidence in overcoming COVID-19	*“I’m a high-risk patient, but just because I’m a dialysis patient does not mean that if I get COVID, it has to end badly. I’ve had the swine flu before; I was isolated then; everyone ran around with masks, but I do not think my body’s defenses are that bad.”* (HD patient, female, age 65)
		Exacerbation of depression because of social isolation	*“I have had depression for a long time, and the symptoms slowly started to get stronger again because of always staying at home and avoiding social contact.”* (HD patient, male, age 25)
		Relative degree of concern over COVID-19 vs. established kidney disease	*“I was more relaxed about [being a high-risk patient] because I knew my kidneys would fail at some point, mainly because they have been bad for a long time. The virus [coronavirus] damages the kidneys even more. It cannot get much worse for me.”* (HD patient, male, age 25)
		Economic impact as concern	*“I was more worried about the economic situation than about myself. Wearing masks and disinfecting your hands from the beginning before going to a shop would have prevented so many unemployed people and companies that had to close.”* (HD patient, female, age 65)
Is Home Dialysis the Best Choice During COVID-19?	Home therapy kept patients away from contact with the hospital, increasing anxiety	Negative impact of isolation on psychological well-being	*“I felt that if this virus infected me, I could die. I experienced generalized anxiety and hysteria. […] Returning home (after self-isolation) was a dangerous decision, but I consciously took this risk because being alone negatively affected me.”* (PD patient, male, age 50)
		Panic about not being transplanted and the negative impact on health	*“I’ve been on a kidney waitlist for three and a half years, and my worst stressful situation was that the transplant program stopped. I am panicking that everything will be postponed, and I will have to wait even longer.”* (PD patient, female, age 57)
	Autonomy of PD treatment as advantage	Confidence in knowing to perform PD and receiving support from the PD team	*“After being on PD for almost three and a half years, I know if I have any problem with PD or something is not working, I can call and go to the unit. I was not afraid that I would not get any information. [...] I think it was easier to dialyze from home and not risk of getting infected somewhere.”* (PD patient, male, age 64)
		Possibility to dialyze at home with strict hygienic conditions	*“I can isolate my entire therapy process from the outer world and have total control over it. I can disinfect the devices and myself. I make sure that the medicines are stored properly and cleanly. I’ve seen doctors and nurses not always disinfecting their hands when they should.”* (PD patient, male, age 50)
Changes in Clinical Routine	Limitations and changes during dialysis routine upset patients and their families	Continuous mask use, not being able to eat during HD sessions considered annoying	*“The mask bothers me because then I cannot breathe or talk. The exhaled air you breathe in is unhealthy because you get tired. So, this is not an ideal solution, but we have no other choice.”* (HD patient, female, age 65)
		Prohibition of visitors at the hospital generated discomfort	*“My wife is so upset because she is not allowed to come into the dialysis unit. I am not afraid because you can keep in touch a bit* via *the internet so that you can see each other.”* (HD patient, male, age 49)
Positive Psychological Elements to Overcome Crisis	Alternative routines to promote well-being	Keeping physically and mentally active	*“I do a lot of sports in the fresh air, and it works luckily quite well. Luckily, I am also allowed to work, and that is how time goes by faster. That is the way I do not get bored.”* (HD patient, male, age 26)
		Learning new skills to communicate with others	*“The opportunity that lockdown has brought me is that only now have I experienced social media and Google. It became clear to me what these tools are for. I exercise with a YouTube video where a trainer exercises with you. This was something I could do at home.”* (PD patient, female, age 62)
	Cultivating positive emotions to transform suffering	The possibility to work, and do sports outdoors as positive aspect	*“I am at the office, and I can ventilate it; it works quite well. Also, in public transport with the mask, I go to only a few stations.”* (HD patient, male, age 26)
	Seeing the glass half full	Family support as positive aspect during crisis	*“I just could not see my daughter and son. However, we were very lucky. After all, we might see each other again because our house has a huge garden. Then we met outdoors, keeping our distance. We could at least see each other.”* (HD patient, female, age 69)
		Trying to adjust to the adverse situation (resilience) psychologically	*“For me, fear is something that inhibits me and makes me insecure. I am trying to reduce that. I always make sure that I can master the current situation as well as possible, which often does not work, but I can get something good out of it every day in my current situation.”* (PD patient, female, age 57)
	Vaccination as hope for high risk patients	Waiting to be vaccinated to visit family	*“It has been a year and a half now that I have not seen my sister. However, I am not going anywhere. First, I want to get the vaccine for safety.”* (HD patient, female, age 54)

#### Concerns about ‘high-risk patient’ status

PD patients did not understand what it meant to be a ‘high-risk patient’ and wanted more information about COVID-19 from their physicians or nurses during consultation. PD patients also stayed at home for longer intervals between appointments at the outpatient clinic. Some patients decided to call the PD team to get information related to COVID-19.

#### Little fear of COVID-19 as a patient on HD

In contrast to some PD patients, HD patients worried more about previous complications of their kidney disease or hospitalizations than about the current pandemic or the possibility of being infected, despite three times per week dialysis sessions.

“The worst thing for me was that the calciphylaxis was supposed to be a deadly disease. I was more afraid then than I am of Corona (COVID-19) now because it was very threatening.” HD patient, female, age 69.

Many HD patients had been previously confronted with critical health situations and had survived. Therefore, these patients were confident they could overcome COVID-19 if infected. Indirect impacts of the pandemic on their daily routine, on their families, or, more generally, on society were of greater importance to HD patients.

“What scared me mostly was not being able to see my kids and friends [if they get infected with COVID-19]”. HD patient, female, age 69.

#### Questions about home dialysis as a better choice during COVID-19

Most PD patients experienced high levels of anxiety and panic, especially previously anxious ones. PD patients were more strict with hygienic measures and intensified disinfection, for example, when PD supplies were delivered at home.

“My husband disinfects what needs to be disinfected; other things are placed immediately underwater. At our house, [cleaning] is excessive”. PD patient, female, age 62.

One PD patient’s decision to self-isolate from his family and social environment to avoid contracting COVID-19 had a negative psychological impact. Another PD patient was distressed by the temporary suspension of the kidney transplantation program. On the other hand, some PD patients saw their autonomy over dialysis and the ability to contact the PD team as advantages.

“I'm much more flexible with the PD, I can do it all at home. I have a family, I have a child, I have a dog, and I go to work. I do it [dialysis] for nine hours on the cycler at night, then I get up, and I'm almost fully fit. It's not like that with hemodialysis, a bigger strain on the body. And as long as my peritoneum plays along as a filter, it's ideal.” PD patient, age 57.

#### Changes in clinical routine

Restrictions of caregivers on visiting patients during HD sessions generated discomfort in patients and their families. There were difficulties in accessing the hospital due to movement restrictions. Separate access to the hemodialysis unit worked adequately, but access through the hospital’s main entrance for diagnostic tests or other interventions was sometimes more difficult. HD patients were not afraid to go to the dialysis unit because “they felt protected there” and had separate access from the rest of the patients in the hospital. Continuous use of face masks and the prohibition of eating during HD upset many patients.

“I think getting tested once a week [for COVID-19 infection] is enough; three or four [patients] in a room is also a good, manageable number. To be all the time with the mask is tedious, but it is necessary. Unfortunately, eating is now almost forbidden or is no longer possible. That was always very practical because time flew.” HD patient, male, age 26.

#### Positive psychological elements to overcome the crisis

During lockdown periods, patients adapted their daily routines to stay physically and mentally active. Patients tried to learn new skills to communicate with others; they performed exercises at home or outdoors.

“Then I thought to myself: I'm home alone, my husband is at work, I don't have anyone who can infect me, so why don't I go out into the garden?” PD patient, female, age 56.

Positive emotions such as resilience, hope, family support, gratitude, and psychological flexibility were more evident in HD patients than in PD patients.

“I just couldn't see my daughter and son. However, we were very lucky. After all, we might see each other again because our house has a huge garden. Then we met outdoors, keeping our distance. We could at least see each other.” HD patient, female, age 69.

HD patients saw vaccination as the possibility that all restrictions could be lifted.

“I'm looking forward to the vaccination when it's finally time so that I can work with contact with people again. People want to get back into gastronomy, to book vacations, to just have plans for something because it's very monotonous and boring, but we have to keep going a bit and it will get better.” HD patient, male, age 26.

Only a few PD patients mentioned coping strategies to improve their well-being and relieve fear and anxiety during the lockdown period.

“The opportunity that lockdown has brought me is that only now have I experienced social media and Google. It became clear to me what these tools are for. I exercise with a YouTube video where a trainer exercises with you. This was something I could do at home.” PD patient, female, age 62.

### Key themes raised by nurses and physicians

**Figure 2:**
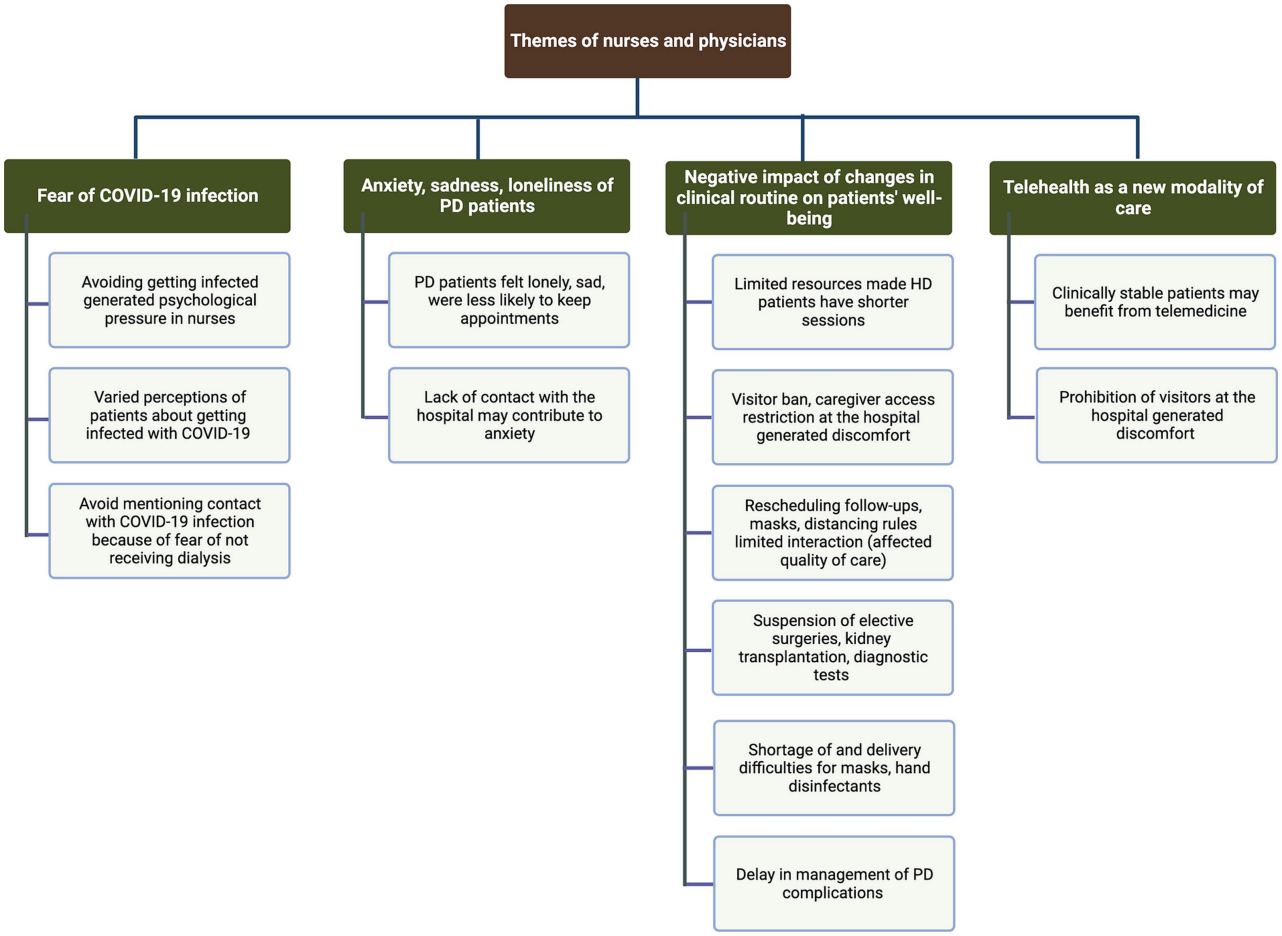
Coding tree identified through content analysis and natural language processing (LDA) of the interviews of nurses and physicians.

Four main themes and subthemes characterized healthcare providers’ experiences: (1) fear of COVID-19 infection (fear of infecting and being infected - as a patient/healthcare professional, fear of patients not receiving dialysis; (2) anxiety, sadness, and loneliness of PD patients (lack of contact with the hospital may contribute to anxiety, loneliness and sadness in PD patients); (3) negative impact of changes in the clinical routine on patients’ well-being (challenges to access dialysis with minimal risk of COVID-19 infection, discomfort with preventive measures and restrictions at the hospital, reduction in patient-physician interaction, prioritization of healthcare resources; shortage of masks and hand disinfectants, difficulties in dealing with dialysis complications); (4) telehealth as a new modality of care (teleconsultation and remote patient monitoring as feasible tools). All topics are presented in a thematic coding tree (Figure 2). Exemplar quotes are included in Table 3.

**Table 3:** Main themes with subthemes and exemplary quotes of nurses and physicians.

Theme complexes	Main themes	Subthemes	Quotation (Participant Role, Sex)
Fear of COVID-19 Infection	Fear of infecting and being infected	Avoiding getting infected generated psychological pressure to nurses	*“My biggest challenge was avoiding COVID-19 spread among patients. We take care of the patient still worrying about doing everything right, taking all measures correctly without forgetting something.”* (Nurse, female)
		Varied perceptions of patients about getting infected with COVID-19	*“The PD patients who came to the PD unit during the lockdown were very relaxed. Sometimes I also have the impression that many older people have thought they had already lived long and enjoyed life, and it had to be over at some point.”* (Physician, female)
			*“There were a few patients who were terribly afraid. The nursing staff can get infected, so it’s important that both patient and staff follow the guidelines.”* (Nurse, female)
	Fear of not receiving dialysis	Avoid mentioning contact with COVID-19 infection	*“Many patients said: ‘What happens to my dialysis if I have contact with COVID-19 and have to go to quarantine”* (Physician, female)
Anxiety, Sadness, and Loneliness of PD Patients	PD patients felt lonely, sad, were less likely to keep appointments	The flexibility of home dialysis made PD patients have longer intervals between appointments	*“HD patients have their dialysis treatment at the hospital and because PD patients have their dialysis at home they often postpone their routine check-ups.”* (Nurse, female)
			*“PD patients experienced loneliness and sadness because their families could not visit them. A HD patient comes to the hospital three times weekly and has a social environment. He/she talks to neighboring patients or the nurse.”* (Nurse, male)
	Lack of contact with the hospital and anxiety	Information from healthcare providers was reassuring for patients	*“After the short talk during a consultation, all questions were answered quite well, and patients were then a little more confident and no longer anxious.”* (Physician, female)
Negative Impact of Changes in Clinical Routine on Patients′ Well-Being	Challenges to access dialysis with minimal risk of COVID-19	Limited resources made HD patients have shorter sessions	*“Patients did not arrive on time to dialysis because the ambulances transported patients individually. As a result, dialysis quality has suffered.”* (Nurse, female)
	Preventive measures at hospital	Visitor ban, caregiver access restriction generated discomfort	*“Patients often needed relatives,* e.g.*, for training or for interpreting, if they do not speak the language. […] Some patients cannot walk alone or need help with many things.”* (Physician, female)
	Reduced patient-doctor interaction affected the quality of care	Rescheduling follow-ups, masks, distancing rules limited interaction	*“There are room dividers between all patients on HD. They cannot even talk to the patient or staff member next to them. All the nurses wear masks and do not talk to the patient unless necessary.”* (Physician, female)
	Prioritization of healthcare resources	Suspension of elective surgeries, kidney transplantation, diagnostic tests	*“Routine annual examinations that all patients have to undergo or appointments for patients without life-threatening conditions were postponed because there was simply no capacity.”* (Nurse, male)
	Shortage of protective material	Shortage of and delivery difficulties for masks, hand disinfectants	*“There were problems with the delivery of products for PD patients. One company ran out of hand disinfectant.”* (Nurse, female)
Negative Impact of Changes in Clinical Routine on Patients′ Well-Being	Difficulties dealing with dialysis complications	Delay in management of PD complications	*“A PD patient with fever was not allowed to come to the outpatient clinic although his (catheter) exit-site might have been infected. The result of the COVID-19 smear did not arrive in time; he was critically ill with sepsis and was admitted to another hospital. […] PD patients cannot really be treated at other hospitals because they do not have the necessary materials.”* (Nurse, female)
Telehealth as a New Modality of Care	Teleconsultation, remote patient monitoring as feasible tools	Clinically stable patients may benefit from telemedicine	*“PD patients have appointments at relatively long intervals, so remote patient monitoring is a good option for stable PD patients because you can monitor dialysis data readout.”* (Physician, male)

#### Fear of infecting and being infected

Physicians mentioned that they did not perceive their patients’ anxiety or fear of infection. Nurses pointed out that they noticed the fear of dialysis patients going to the hospital for appointments.

“The patients were certainly afraid. They either have to use public transportation or they have to come by ambulance. They didn't want to come to the hospital at all.” Nurse, female.

Some physicians did not proactively discuss patients’ higher risk in case of being infected with COVID-19 “because patients already knew they were risk patients.” Other physicians only talked about risks of COVID-19 infection if patients asked directly about any special or additional protective measures, such as immunosuppressive treatment in the context of kidney graft failure. However, “very relaxed patients” were among those who felt they had already lived long enough. As this group of patients was not afraid of COVID-19 infection, they came to the hospital for follow-up, whereas more anxious PD patients preferred to stay at home for fear of being infected by healthcare providers.

Nurses also mentioned that avoiding getting infected with COVID-19 generated psychological pressure on them because they did not want to infect frail dialysis patients.

#### Fear of patients not receiving dialysis

Healthcare providers suspected that HD patients avoided mentioning any symptoms or contact with a COVID-19 infection due to fear of not receiving their scheduled dialysis. Confirmation of a COVID-19 infection would lead to the performance of HD at another hospital designated for COVID-19 patients, or HD would be performed during the night shift at the patient’s usual dialysis unit.

“Many patients said: 'What happens to my dialysis if I have contact with COVID-19 and have to go to quarantine?” Physician, female.

#### Anxiety, loneliness, and sadness of PD patients

Nurses, rather than physicians, noticed that PD patients were canceling appointments at the hospital more frequently than HD patients because they had the option to do so.

“I noticed that PD patients are more likely to postpone their appointment than HD patients. HD patients come to their appointment because they have treatment there, and because PD patients have their dialysis at home, they often postpone the appointments for routine check-ups.” Nurse, female.

Home treatment made some PD patients feel safe but, at the same time, more isolated and lonely.

“When PD patients are at home with their family, they feel safe, but when they have a problem, then they have to go to the hospital and have to deal with others because they have to sit in a room with other patients.” Nurse, male.

In contrast, HD patients had their “fixed social point” at the hospital and interacted with other patients and staff members. Some nurses and physicians mentioned that some married PD and HD patients experienced divorce or separation during the pandemic.

“Some patients are socially lonely due to the lockdown. Dialysis patients usually feel isolated, even before the pandemic. I've already heard from two or three patients that they feel lonely after (relationship) breakups, and I then offered them psychological care.” Nurse, female.

#### Lack of contact with the hospital and anxiety in PD patients

Physicians suspected that lack of contact with the PD team made PD patients feel more anxious, with more concerns related to the implications of COVID-19 for their clinical or personal situation. In contrast, the frequent contact of HD patients with healthcare providers and the hemodialysis setting contributed to their less prominent fear of coronavirus infection related to treatment.

“Other PD patients were more anxious and moved their appointments to longer intervals; they went to the laboratory and then sent their results and talked with us via telephone because they did not want to come to the hospital. […] And then others were alone and isolated.” Physician, female.

#### Challenges to access dialysis with minimal risk of COVID-19 infection

Nurses mentioned that HD patients were at elevated risk of COVID-19 exposure during their ambulance transport to and from dialysis sessions. More autonomous PD patients traveled by car or taxi to avoid virus exposure. HD unit schedules were busy, and ambulance availability was limited because each HD patient was transported separately to minimize exposure among patients. The quality of dialysis was affected by occasional delays in session start times and occasional reductions in dialysis duration. Interestingly, frequent contact with other patients and medical staff did not worry HD patients.

“The HD patients had confidence that preventive measures would be followed at the ambulances to transport them to the dialysis unit, where they had completely separate access.” Nurse, male.

#### Discomfort with preventive measures and restrictions at the hospital

Triage sites for screening patients were placed at the hospital and dialysis unit entrances, delaying patients’ access to the hospital. Caregivers and family members were not allowed to join patients. Healthcare providers felt this was especially burdensome for PD patients, who frequently needed assistance from their spouse.

#### Reduction in patient-physician interaction

Communication barriers increased as the frequency of medical rounds decreased, and facemask use increased, while room dividers were placed between HD patients during HD sessions. Some physicians tried to maintain physical distance from patients during consultations.

“Personally, patients have not asked me about COVID-19 information. At the beginning of the whole story, there was very little doctor-patient contact because we did not go on [clinical] rounds. If there was a contact, it was rather with the nurses.” Physician, male.

#### Prioritization of healthcare resources

There was greater concern about health deterioration because of the deferral of kidney transplantation and the possibility of future changes in the dialytic regimen.

“There have been patients who, unfortunately, have their selective surgeries postponed, and the problem has become bigger over time. (...) For example, patients with problems in the musculoskeletal system have also waited longer and thus had to take more painkillers until they have their surgery.” Physician, female.

Routine diagnostic tests were canceled, including periodic tests for kidney transplant eligibility. Physicians mentioned patient concerns about registration on the transplant waitlist.

“The kidney transplant was a major concern for all listed patients. Many PD patients know that the transplantation program has stopped for a time. Some have asked what happens if there is a (kidney) offer during that time, if the waiting period starts over again.” Physician, female.

One patient waiting for kidney transplantation chose removal from the transplant waitlist due to increased risk of severe COVID-19 infection during post-transplant immunosuppression.

#### Shortage of masks and hand disinfectants

Healthcare providers mentioned shortages of masks and hand disinfectants at the beginning of the pandemic. Although nurses initially feared problems with the corporate delivery of PD solutions due to border restrictions during the first COVID-19 wave, supplies of PD solutions remained adequate.

#### Difficulties in dealing with dialysis complications

Febrile patients with possible PD-associated problems (e.g. peritonitis) could not visit the PD unit directly because a COVID-19 screening was needed.

“If PD patients have a problem and are COVID-19 positive, they cannot come to the PD unit. If they have problems with the machine (cycler) or the (cycler card) or inflow catheter problems, that's certainly a bit difficult logistically to send a nurse to their homes.” Physician, male.

A physician mentioned difficulties for patients to access the outpatient PD clinic or the HD unit when reporting specific symptoms of COVID-19 at triage.

“To what extent are these symptoms now dependent on dialysis or uremia (fatigue, dizziness, dyspnea, or headaches), or are these related to COVID-19?” Physician, female.

Moreover, those hospitals designated for dialysis of COVID-19 patients were not equipped with devices from all PD providers used by patients, resulting in difficulties to organize treatment if they were admitted to these other hospitals.

“The patient was admitted to another hospital because of bacterial pneumonia (initially suspected to be a COVID-19 infection). His wife brought him the [cycler] machine, which weighs about 13 kilograms, and a box that weighs 10 kilograms with the dialysate bags he needed.” Nurse, female.

#### Telehealth as a new modality of care

Physicians stated that teleconsultation and remote patient monitoring devices could help care for fragile patients with advanced age or significant comorbidities.

“I think telemedicine would be ideal if we could implement it (in our PD outpatient clinic) if there is an increase in cases or a new virus comes because PD patients then have a [cycler software] card. If it can be read, they do not have to go to the hospital” Physician, female.

Nevertheless, nurses thought that providing patients with exclusively virtual training in practical skills at PD initiation might not be adequate.

### Similar and distinctive themes between groups of participants

**Figure 3:**
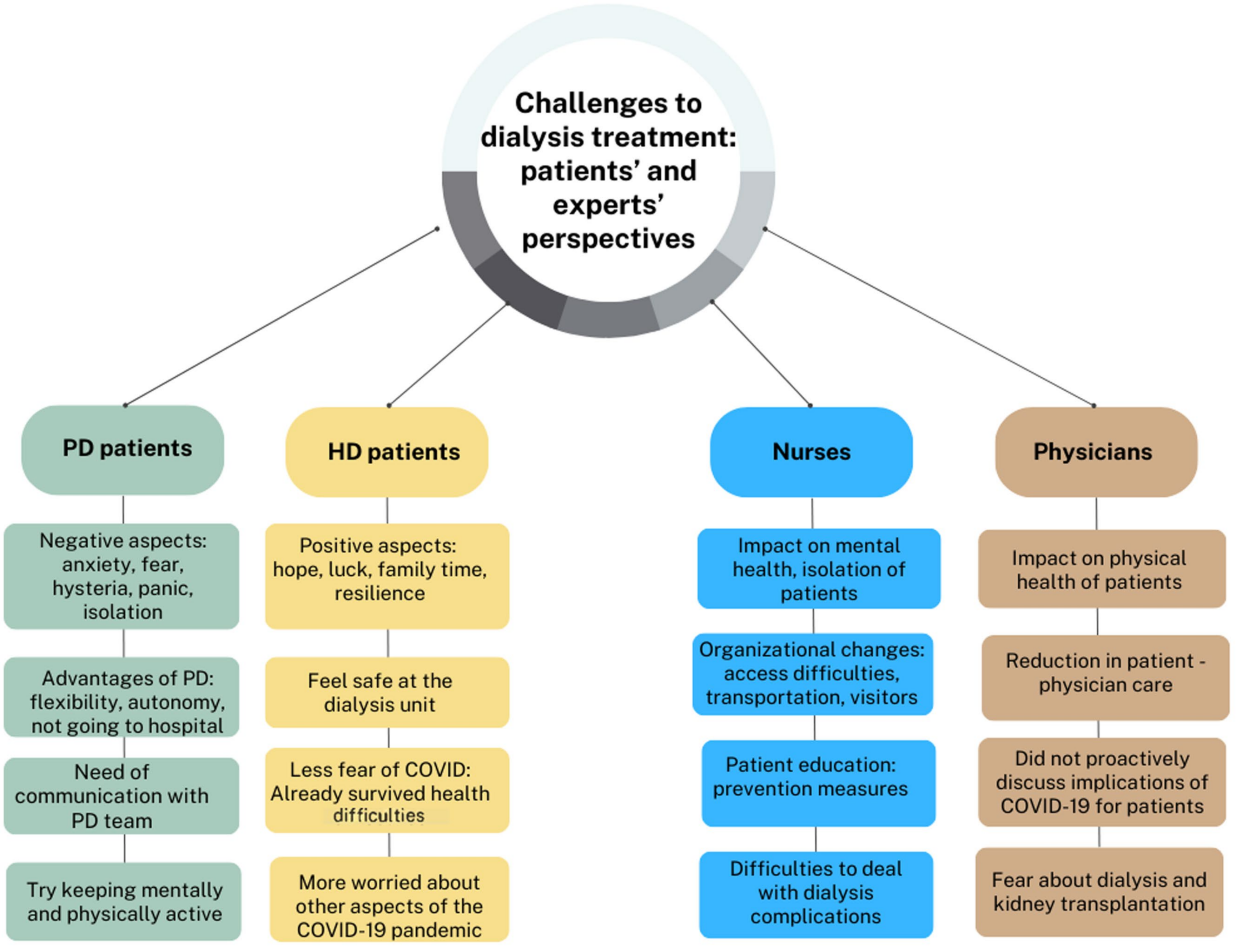
Distinctive themes identified in the four groups of participants.

We summarize the most distinctive themes mentioned by patients and healthcare providers in Figure 3.

In the case of PD patients, fear of being infected, social isolation, and concern (e.g., about not being transplanted) were frequent themes that emerged from the interviews. Conversely, in the case of HD patients, words frequently mentioned related to spending more time with family, hope that the crisis would be over (with vaccination), and trust in their dialysis team. We identified a greater fear of going out and more anxiety in PD patients. Negative terms were more frequently mentioned by PD patients (“hysteria,” “panic,” “danger,” “fear,” and “lonely”).

HD patients focused mostly on positive aspects of the pandemic. Positive terms such as “lucky” (e.g., if they were retired, it was easier for them to resist staying at home, or if they worked, they said going out to work was a distraction); “hope” (when talking about the returning to “normality” and vaccination) were more frequently mentioned.

Physicians and nurses agreed that PD patients were younger, more active, and more autonomous. Conversely, most of the HD patients were more fragile, with more comorbidities than PD patients, which could increase their fear of going to the hospital for dialysis. However, younger HD patients were less afraid of COVID-19 infection, and some older patients were more worried about their family getting infected or complications of their kidney disease or other comorbidities.

Nurses mentioned aspects related to the social isolation of patients and organizational difficulties more frequently in the interviews (ambulance transportation, visitors ban, difficulties at the hospital entrance, and patient education on the prevention of COVID-19 infection). Physicians more frequently mentioned aspects related to disruptions in health care with negative impacts on patients’ health (suspension of the transplantation program, suspension of elective surgical interventions, cancelation of appointments, hemodialysis night shift for COVID-positive patients).

Aspects related to changes in the mental health of dialysis patients (e.g., increased anxiety, fear, worry, isolation) were more frequently mentioned by nurses. Nurses and physicians agreed that PD patients were more anxious about attending the outpatient clinic and that HD patients were also scared of being infected with the coronavirus when going to the hospital. Nurses and physicians noted increased anxiety in dialysis patients when the transplantation program was temporarily stopped.

Concerning discussion of preventive measures for COVID-19 with patients, nurses mentioned that they emphasized hygiene education of dialysis patients. Physicians reported that although they answered patients’ questions, they did not discuss preventive measures and hygiene with all patients.

## Discussion

This qualitative study evaluated challenges faced by in-center HD and PD patients after the COVID-19 outbreak and compared their perspectives with those of their healthcare providers. Higher anxiety levels, fear of infection, and social isolation depended on dialysis modality and pre-pandemic mental health status. PD patients appeared more profoundly impacted psychologically, probably due to a lack of contact with the group dialysis setting. We found a relevant knowledge gap between patients and healthcare providers, possibly reflecting reduced patient-physician interaction. Many physicians did not proactively assess aspects related to COVID-19 during consultations, whereas nurses more often perceived patients’ psychological issues. Healthcare institutions should provide access to reliable and updated information about COVID-19 to address dialysis patients’ concerns.

Chronically ill patients exhibit a high risk for adverse clinical outcomes of COVID-19, with psychological implications (Brown et al., 2020; Lee et al., 2020; Bonenkamp et al., 2021; Arevalo Iraheta et al., 2022). Current recommendations to increase home dialysis during the pandemic cannot be confirmed (Brown et al., 2020; Cozzolino et al., 2021). Our findings reveal that lack of contact between PD patients with the hospital and the PD team was associated with heightened concern about the impact of COVID-19 on the future of their dialysis or kidney transplantation. We noted that PD offered patients the advantages of home dialysis, with extended intervals between routine follow-ups, a pattern unavailable to most in-center HD patients. However, PD patients more frequently highlighted negative aspects of the pandemic, including fear of being infected, social isolation, and concern about not being transplanted.

In contrast, HD patients focused more frequently on positive aspects, like having more time to spend with family, hope that the crisis would come to an end (with the advent of vaccination), and trust in their dialysis team. Especially anxious dialysis patients and their caregivers had a greater need for reliable information related to COVID-19 (Arevalo Iraheta et al., 2022; Sousa et al., 2022). Many dialysis patients feared COVID-19 due to their high-risk status (Sousa et al., 2021; Arevalo Iraheta et al., 2022). PD patients frequently canceled outpatient clinic appointments. Differences in anxiety levels and dealing with fear of infection were also reflected in the intensity of measures adopted by patients, such as hypervigilance with hygiene (Arevalo Iraheta et al., 2022) or self-isolation, more frequently among patients with previous mental health conditions. One PD patient even moved away from his family into “prophylactic quarantine” for several months, with negative psychological consequences. On the other hand, HD patients adhering to their dialysis schedule did not fear infection during transport or at the dialysis unit, in contrast to the findings of another study (Lee et al., 2020). The change in personal routines (e.g., not meeting family or friends), complications associated with advanced kidney disease (e.g., calciphylaxis) and the economic impact of COVID-19 on society generated more significant concern in HD patients than the risk of infection.

Our findings showed that PD patients mentioned aspects of social isolation more frequently than did HD patients, especially those living alone. During Austrian lockdowns in March and November 2020, many patients had stricter quarantines with extended periods of isolation, affecting their mental health (high levels of anxiety, depression) and even disrupting personal relationships (separation, divorce). Patients with a partner, those living in a flat-sharing community or a house with a garden, and employed patients were more likely to tolerate lockdown. Many HD patients did not perceive a change in social isolation because there was already loose contact with the family before the pandemic or patients were already restricted due to the rigid three-times-per-week dialysis regimen or due to other illnesses (e.g., amputations). Importantly, living alone has been perceived as a barrier to patients choosing or being offered treatment with PD in areas without assisted PD programs (Brown, 2008). Thus, PD patients often have a better social environment. Nonetheless, our study showed that PD patients felt isolated because of lack of contact with the PD team. In contrast, the three-times-weekly HD treatment represents an opportunity to maintain social contact in HD patients (Lee et al., 2020; Bonenkamp et al., 2021; Malo et al., 2022). However, some depressed HD patients noted worsening symptoms due to isolation from family and friends during lockdown (Lee et al., 2020).

Deferral of elective surgical interventions, diagnostic procedures, and implementation of longer intervals between PD unit appointments could have harmed patients’ physical and mental health (Guha et al., 2020; Arevalo Iraheta et al., 2022). We also confirmed that suspending the kidney transplantation program led to panic and high stress levels in some patients, who feared deterioration in their health in the face of increased uncertainty about future transplantation (Guha et al., 2020). On the other hand, one patient rejected future transplantation due to concerns about increased COVID-19 risk when immunosuppressed. Therefore, patients should have access to psychological support and informed decision-making.

Assessment of dialysis-related complications (e.g., peritonitis and catheter problems) was impeded by restriction measures in the presence of symptoms suggesting COVID-19 infection. Dyspnea, headache, dizziness, or fatigue are common uremic symptoms in dialysis patients that are not specific to COVID-19. PD patients with confirmed COVID-19 infection were transferred to other centers, where PD equipment or cyclers of only one provider type were available. This resulted in difficulties for some patients in performing their usual treatment regimen. Some HD patients avoided mentioning symptoms possibly related to COVID-19, fearing that they would not receive regular dialysis treatment or be transferred to another center.

Healthcare professionals in our study reported that using facemasks, distancing between patients and healthcare providers, and teleconsultation represented barriers to communication, leading to less frequent patient-physician interaction. Healthcare professionals further stated that regular telemedical or telephone consultation is a feasible option to minimize in-person interaction for outpatients (not on dialysis) and high-risk or stable PD patients (Cozzolino et al., 2020). The use of telemedicine for in-center and home-hemodialysis patients has promoted independence and satisfaction with patient care (Antoun et al., 2021).

Interestingly, positive psychological elements such as resilience, hope, gratitude, and social support were more evident in HD patients than in PD patients. As noted by others, family members and friends played an important supportive role for patients (Sousa et al., 2021, 2022). Resilience during the COVID-19 pandemic was greater in HD patients with more prolonged dialysis vintage (Malo et al., 2022). Patients who had already survived critical health experiences had more psychological resources and flexibility to cope with the long pandemic than healthier patients not exposed to these situations. In contrast to other findings (Xia et al., 2021), we noted higher confidence in overcoming COVID-19 in HD patients than in some PD patients. Dialysis patients adapted their routine during lockdown to promote physical and mental well-being (Mosor et al., 2021). Psychological flexibility, the ability to adjust cognition and behavior according to personal needs, is related to lesser anxiety and depression, explaining resilience and preservation of mental health (Israelashvili, 2021; Pellerin et al., 2022).

A strength of our study is its status as the first qualitative study comparing dialysis patients (PD and in-center-HD patients) and their healthcare providers. Our study gathered information on how healthcare providers perceived patients’ views during the COVID-19 pandemic and, on the other hand, what patients themselves expressed. Our study also implemented a thematic content analysis supported by a natural language processing technique, a new methodological approach not previously applied in this context.

Among the limitations of our study is its exploratory nature, with a small sample size (n = 28) from a single clinical center. Thus, validation will require larger dialysis populations distributed internationally. Our selection of participants was purposive. This recruitment method may have generated a selection bias and may not reflect the experiences and challenges of patients or healthcare providers who did not participate in these interviews. The time span of our interviews ranged from March 2020 to February 2021. Understanding of patients being at high risk and requiring information about COVID-19 may have changed over time. We also did not assess personal experiences about testing positive for COVID-19 of patients or their families, which may limit the generalizability of our study.

### Implications for future strategies

The general population’s fear of COVID-19 infection has decreased over time. Since patients have become less adherent to anti-COVID-19 measures, the dialysis team should intervene and reinforce information about and preventive measures against COVID-19. In the event of recurrent waves of COVID-19 or future pandemics, assessment of dialysis patients should incorporate more frequent psychological evaluation. Selection or modification of dialysis modality after the COVID-19 pandemic outbreak should include a careful analysis of individual psychosocial conditions by the dialysis team. Structural improvements at the dialysis units (redesign/enlargement of waiting and entrance areas) and more extensive teleconsultation implementation are needed to manage future pandemics. Our findings should serve as a basis for developing more personalized guidelines or strategies for dialysis facilities during pandemics which should be adapted to individual dialysis patients’ treatment goals and needs.

## Data availability statement

The original contributions presented in the study are included in the article/Supplementary material, further inquiries can be directed to the corresponding author.

## Ethics statement

The study was approved by the intra-university Data Protection Committee and the Local Ethics Committee of the Medical University of Vienna (study protocol EK 1725/2020). The studies were conducted in accordance with the local legislation and institutional requirements. The participants provided written informed consent to participate in this study.

## Author contributions

KOF and AV developed the concept and design of the study, had full access to all data, and took responsibility for the integrity and accuracy of data and subsequent analysis. KOF, AV, and VR performed data collection and contributed to the interpretation of data. KOF, VR, and TS conducted interviews analysis. KOF drafted the initial manuscript. KOF, TS, SA, VR, and AV did a critical revision of the manuscript for important intellectual content. All authors contributed to the article and approved the submitted version.
