# Supplementary material for: Challenges to dialysis treatment during the COVID-19 pandemic: a qualitative study of patients’ and experts’ perspectives
Source: Front Psychol. 2023 Nov 15;14:1185411. doi: 10.3389/fpsyg.2023.1185411 (PMC10686285; doi:10.3389/fpsyg.2023.1185411)
Supplement: Supplementary file 1 [file Data_Sheet_1.docx]

**Challenges to dialysis treatment during the COVID-19 pandemic: a qualitative study of patients’ and experts’ perspectives**

Krystell Oviedo Flores ^1^, Tanja Stamm ^2,3^, Seth L. Alper ^4^, Valentin Ritschl ^2,3 †,^ Andreas Vychytil ^1†^

^1^Division of Nephrology and Dialysis, Medical University of Vienna, Vienna, Austria; ^2^Institute of Outcomes Research, Center for Medical Data Science, Medical University of Vienna, Vienna, Austria; ^3^Ludwig Boltzmann Institute for Arthritis and Rehabilitation, Vienna, Austria; ^4^Division of Nephrology, Beth Israel Deaconess Medical Center and Department of Medicine, Harvard Medical School, Boston, MA, United States of America.

†Both authors contributed equally and share last authorship

**Table of contents of supplementary material**

[Supplementary Table S1. Interview guides for dialysis nurses and nephrologists 2](#_Toc144720576)

[Supplementary Table S2. Interview guides used for peritoneal dialysis and hemodialysis patients 3](#_Toc144720577)

[Supplementary Table S3. COREQ (COnsolidated criteria for REporting Qualitative research) checklist to enable the reader to check the quality of this paper quickly 4](#_Toc144720578)

Supplementary Table S1. Interview guides for dialysis nurses and nephrologists

| Topic Details Prompts  Prompts | | |
| --- | --- | --- |
| **Introduction, ice-breakers, informed consent** | Thank you for seeing me today and offering to take part in this study. I would like first to outline the study so that you are able to decide whether you wish to proceed further (recap information sheet).  I would like to invite you to share your ideas and experiences related to you treat during this COVID-19 pandemic. | - Any difficulties, fears or problems experienced by the dialysis patients |
| **Research goals** | This study is intended to help improve actions taken in response to the novel coronavirus pandemic.  1) What impacts and consequences do the restriction measures during COVID-19 pandemics have on the dialysis treatment?  2) What did you perceive as “negative” or not well managed at the dialysis units and follow-ups? | - How does the new COVID-19 pandemics affect your dialysis patient’s life - in what ways? - What do you think is the most challenging patients have to deal with - why? |
| **Prevention -own behavior** | Prevention measures (hand washing, avoid touching face, disinfectants, stay at home, physical distancing, face mask, avoiding crowds, self-quarantine, not seeing family, friends) | - What are preventive measures you are applying at the dialysis unit to avoid infection with SARS/CoV-2/ Coronavirus? (home/at hospital/during routine follow-up) |
| **Treatment**  **Follow up**  **Appointments**  **Surgeries** | Regime modifications, problems with appointments (related to transplantation), problems with follow-ups? | - Was the dialysis regime of patients modified during COVID-19? -why? - Any difficulties with patients’ follow-up at the hospital? - Opinion of telemedicine/telephone follow-up? |
| **Problems at the hospital** | Access to dialysis unit, no possibility of family/visitors | - Difficulties of patients to access the hospital/dialysis unit? - How does the absence of visits or caregiver at the hospital affect patients? |
| **Worries** | Level of worry (related to losing a loved one, being a risk patient, health system overload, dialysis supply, dialysis adequacy) | - Were patients scared about their personal risk of severe infection? - Differences perceived among PD/HD patients? - Changes perceived in mental health during quarantine? - Problems with dialysis supply of fluids, disinfection solutions for PD patients? |
| **Sources of information regarding risks of virus infection to dialysis patients** | Who from? | - Have you addressed the risk of infection with SARS-CoV2/Coronavirus with your patients? - Any other sources of information for patients? |
|  | Reaction | - Reactions of patients after receiving this information? |
| **Trust in**  **institutions**  **(perceptions)** | Trust in ability of stakeholders to handle situation (own doctor, company delivering dialysis product, local health department, Ministry of Health, National Health System, public transportation) | - Do patients say they feel safe at dialysis unit (not getting infected) - Reactions of patients towards transplantations list, appointments changed? |
| **Resilience**  **(perceptions)** | Perceptions related to coping with stress and recovering. | - What are your strategies to cope with stress of your patients related to the COVID-19 pandemics? |
| **Conclusion** | Sum up what has been discussed, mention the positive aspects. The participant will be told that if he or she wants to withdraw from the study, their data will not be used. | - Is there anything important to you we haven't mentioned? |

Supplementary Table S2. Interview guides used for peritoneal dialysis and hemodialysis patients

| Topic Details Prompts  Prompts | | |
| --- | --- | --- |
| **Introduction, ice-breakers, informed consent** | Outline of the study to let patient decide whether to proceed further (recap information sheet).  Sign consent form × 2 (one for participant and information sheet, one for interviewer). | Ideas and experiences related to any difficulties, fears or problems experienced with your dialysis treatment during this COVID-19 pandemic.  Aim of study: to improve actions taken in response to the novel coronavirus pandemic. (COVID-19) |
| **Research goals** | I have a list of topics that I want to address.  1) What impact and consequences do the restriction measures during COVID-19 pandemics have on your dialysis treatment?  2) What did you perceive as “negative” or not well managed with your dialysis and follow-ups? | How does the new COVID-19 pandemics affect your life - in what ways?  What is the most challenging to deal with - why and how do you cope with it? |
| **Prevention - own behavior** | Prevention measures (hand washing, avoid touching face, disinfectants, stay at home, physical distancing, face mask, avoiding crowds, self-quarantine, not seeing family, friends) | Preventive measures you are applying to protect yourself against the SARS-CoV-2/ Coronavirus infection?  At home or at hospital for treatment/control |
| **Treatment**  **Follow up**  **Appointments**  **Surgeries** | Regime modifications, Problems with appointments (related to transplantation), problems with follow-ups? | Was your dialysis regime modified during COVID-19? -why?  Difficulties with your follow-up at the hospital? Worried about longer intervals/appointments to transplantation?  Feelings towards possibility of calling to the dialysis unit in case of questions related to COVID-19? |
| **Problems at the hospital** | Access to dialysis unit, no possibility of family/visitors | Any difficulties accessing the hospital/dialysis unit?  How does the absence of visitors or your support person at the hospital affect you? |
| **Worries** | Level of worry (related to losing a loved one, being a risk patient, health system overload, dialysis supply, dialysis adequacy) | Worried about: receiving supply at home? (masks, disinfectants, PD solutions), your dialysis treatment adequacy? (absence of blood controls)  Mental health during these days of quarantine? Sad/depressive, Anxious, Problems sleeping |
| **Sources of information regarding risks of virus infection to dialysis patients** | Who from? | Have doctors/other health professional or anyone else talked about the risk of a SARS-CoV-2/ Coronavirus infection?  Any other sources of information? |
|  | Reaction | How did you feel after receiving this information? |
|  | Other information wanted | Any other information you wanted, or that you have tried to access? |
|  |  | If so, what type of information, from whom would you prefer it? |
| **Trust in**  **institutions**  **(perceptions)** | Trust in ability of stakeholders to handle situation (own doctor, company delivering dialysis product, National Health System, public transportation) | Do you feel safe at the dialysis unit? |
| **Resilience**  **(perceptions)** | Perceptions related to coping with stress and recovering. | What are your strategies to cope with stress related to the COVID-19 pandemic? |
| **Lifting restrictions (pandemic transition phase)** | Perceptions related to lifting restrictions (in general, different restrictions depending on age, geography) | How do you feel about the restrictions been lifted?  obligatory face mask  Own wish to maintain restrictions |
| **Conclusion** | Sum up what has been discussed, mention the positive aspects.  The participant will be told that if he or she wants to withdraw from the study, their data will not be used. | Is there anything important to you we haven't mentioned? |

Supplementary Table S3. COREQ (COnsolidated criteria for REporting Qualitative research) checklist to enable the reader to check the quality of this paper quickly

| **Topic** | **Item no.** | **Guide Questions/’Description** | **Reported in section** |
| --- | --- | --- | --- |
| **Domain 1: Research team and reflexivity** | | | |
| **Personal characteristics** | | | |
| Interviewer/facilitator | 1 | Which author/s conducted the interview or focus group? | The first author conducted all interviews. This is reported in the methods section:  *To minimize reporting bias, all interviews were performed by K.O.F. (female, MD, MSc, and candidate for the Ph.D. degree), who was uninvolved in the patients' care and otherwise unrelated to the patients.* |
| Credentials | 2 | What were the researcher’s credentials? E.g. PhD, MD |  |
| Occupation | 3 | What was their occupation at the time of the study? |  |
| Gender | 4 | Was the researcher male or female? |  |
| Experience and training | 5 | What experience or training did the researcher have? | The interview guidelines were adapted by T.S, and the data-analysis was primarily reviewed by V.R. These colleagues have extensive experience in the field of qualitative research (TS, VR). This is reported in the methods section*:*  *An experienced qualitative researcher (T.S.) reviewed and adapted the interview guide. (…) An additional researcher (V.R.) with extensive experience in qualitative research reviewed the results.* |
| **Relationship with participants** | | | |
| Relationship established | 6 | Was a relationship established prior to study commencement? | There was no relationship between the interviews and the participants before the study. This is reported in the methods section:  *To minimize reporting bias, all interviews were performed by K.O.F. (female, MD, MSc, and candidate for the Ph.D. degree), who was uninvolved in the patients' care and otherwise unrelated to the patients.* |
| Participant knowledge of the interviewer | 7 | What did the participants know about the researcher? e.g. personal goals, reasons for doing the research | The participants were informed comprehensively about the goals and the content of the study. They had to give oral and written consent to be included in this study. This is reported in the methods section, but also mentioned in the ethics declaration:  *Patients were informed about the study and invited to participate by phone or during a routine hospital visit. A single one-on-one interview by appointment at the participant's choice was conducted at our center. All participants provided written informed consent. The study was performed according to the principles of the Declaration of Helsinki. Approval was granted by the intra-university Data Protection Committee and the Local Ethics Committee of the Medical University of Vienna (study protocol EK 1725/2020).* |
| Interviewer characteristics | 8 | What characteristics were reported about the interviewer/facilitator? e.g. Bias, assumptions, reasons and interests in the research topic |  |
| **Domain 2: Study design** | | | |
| \| **Theoretical framework** \| \| --- \| | | | |
| Methodological orientation and theory | 9 | What methodological orientation was stated to underpin the study? e.g. grounded theory, discourse analysis, ethnography, phenomenology, content analysis | We used a meaning condensation content analysis, supported by a natural language processing technique, as reported in the methods section:  *Qualitative content analysis is suitable for our exploratory approach to understanding behaviors associated with a human condition in different contexts and perceived situations. (…) We conducted an inductive thematic analysis of qualitative data to discover topics describing patients' experiences and perceptions of their dialysis during the COVID-19 pandemic, followed by a modified meaning condensation form. Data were divided into meaning units (defined as specific text parts, either a few words or sentences with a common meaning) summarized in one or more concepts. Associated concepts were grouped, and a scheme of lower- and higher-level concepts was developed. Lower-level concepts share the attributes of the higher-level concepts but are more specific. (…) Additionally, we used a natural language processing technique called Latent Dirichlet Allocation (LDA) to support the thematic analysis. LDA is an unsupervised, generative, probabilistic topic modeling technique that extracts meanings from a pre-defined number of topics/concepts.* |
| **Participant selection** | | | |
| Sampling | 10 | How were participants selected? e.g. purposive, convenience, consecutive, snowball | We selected patients and health care providers by convenience sampling. This is reported in the methods section:  *Patients were selected using convenience sampling, including a broad spectrum of age, years of dialysis treatment, and comorbidities. Patients with severe cognitive impairment were excluded. Also included were seven nephrologists or nephrology trainees and seven nurses with experience in both dialysis modalities, with a wide range of seniority and experience.* |
| Method of approach | 11 | How were participants approached? e.g. face-to-face, telephone, mail, email | All patients were approached via telephone or personally invited to participate in the interviews. Interviews were performed face-to face. This is reported in the methods section:  *Patients were informed about the study and invited to participate by phone or during a routine hospital visit. A single one-on-one interview by appointment at participant’s choice was conducted at our center.* |
| Sample size | 12 | How many participants were in the study? | Information about the sample and sample size is provided in the methodology/results sections:  *We recruited fourteen patients in maintenance dialysis, seven treated with in-center HD and seven treated with PD. (…)Also included were seven nephrologists or nephrology trainees and seven nurses with experience in both dialysis modalities, with a wide range of seniority and experience.* |
| Non-participation | 13 | How many people refused to participate or dropped out? Reasons? |  |
| **Setting** | | | |
| Setting of data collection | 14 | Where was the data collected? e.g. home, clinic, workplace | Interviews were conducted face-to-face interviews at the PD out-patient’s clinic or at the HD unit, according to the preferences of the participants. All interviews were conducted one-on-one. The following paragraph is reported in the methods section:  *A single one-on-one interview by appointment at participant’s choice was conducted at our center.* |
| Presence of non-participants | 15 | Was anyone else present besides the participants and researchers? |  |
| Description of sample | 16 | What are the important characteristics of the sample? e.g. demographic data, date | The baseline characteristics of the sample are reported in Table 1. |
| **Data collection** | | | |
| Interview guide | 17 | Were questions, prompts, guides provided by the authors? Was it pilot tested? | *Based on an anonymous questionnaire developed to improve routine care of dialysis patients at the outbreak of the COVID-19 pandemic, we created a topic guide that covered aspects related to sources of information, preventive measures against COVID-19, problems with consumables (e.g. hand disinfectant, PD solutions), patients’ experience at the dialysis unit, and psychological aspects (Supplementary Tables 1, 2).* |
| Repeat interviews | 18 | Were repeat interviews carried out? If yes, how many? | The interviews were not repeated since we thought that there will be no further benefit repeating the interviews. This is reported in the methods section:  *A single one-on-one interview by appointment at participant’s choice was conducted at our center.* |
| Audio/visual recording | 19 | Did the research use audio or visual recording to collect the data? | Interviews were all audiotaped and transcribed verbatim. This is reported in the methods section:  *Semi-structured interviews conducted at the center were audio-recorded and transcribed verbatim.* |
| Field notes | 20 | Were field notes made during and/or after the interview or focus group? |  |
| Duration | 21 | What was the duration of the interviews or focus group? | This is reported in the result section:  *The average interview length was 66 minutes (with a range of 46 to 106 minutes).* |
| Data saturation | 22 | Was data saturation discussed? | All eligible patients who agreed to participate in this study were included. |
| Transcripts returned | 23 | Were transcripts returned to participants for comment and/or correction? | We decided not to return the transcripts to the participants for comments and/or corrections. However, three researchers (K.O.F., A.V. and V.R.) reviewed data analysis to ensure that the results covered patients’ views (reported in the methods section):  *An additional researcher (V.R.) with extensive experience in qualitative research reviewed the results.* |
| **Domain 3: analysis and findings** | | | |
| **Data analysis** | | | |
| Number of data coders | 24 | How many data coders coded the data? | The data coding and analysis was primarily done by K.O.F. and A.V. Two researchers reviewed all the data (in the methods section):  *All transcripts were carefully read and checked for accuracy. Coding was performed independently by K.O.F and A.V.(…) Extensive discussion among V.R., A.V. and K.O.F. resolved disagreements to reach consensus through the triangulation technique.* |
| Description of the coding tree | 25 | Did authors provide a description of the coding tree? | Coding trees are provided in Figures 1 and 2, where the main themes and subthemes are presented separately for patients and for healthcare providers. |
| Derivation of themes | 26 | Were themes identified in advance or derived from the data? | *As reported in the methods section, the data analysis was inductive.*  *We conducted an inductive thematic analysis of qualitative data to discover topics describing patients' experiences and perceptions of their dialysis during the COVID-19 pandemic, followed by a modified meaning condensation form.(…)* *Additionally, we used a natural language processing technique called Latent Dirichlet Allocation (LDA) to support the thematic analysis* |
| Software | 27 | What software, if applicable, was used to manage the data? | We used the Atlas.ti version 8 for the analysis and coding, R was used to conduct the natural language processing (LDA), and GraphPad Software was used for descriptive statistics of patients characteristics, all reported in the methods section. |
| Participant checking | 28 | Did participants provide feedback on the findings? | The participants did not provide feedback on the findings |
| **Reporting** | | | |
| Quotations presented | 29 | Were participant quotations presented to illustrate the themes/findings? Was each quotation identified? e.g. participant number | Quotations are included in the results section and in Tables 2 and 3. Each quotation was indexed using participant group, gender, and age in the case of patients. For example:  *“Then I thought to myself: I'm home alone, my husband is at work, I don't have anyone who can infect me, so why don't I go out into the garden?” PD patient, female, age 56.* |
| Data and findings consistent | 30 | Was there consistency between the data presented and the findings? | We endeavored to ensure consistency between the data presented and the findings by using quotes to support our interpretations/findings. Please see the results section, Tables 2 and 3. |
| Clarity of major themes | 31 | Were major themes clearly presented in the findings? | We presented our themes in coding trees in Figures 1 and 2, and in Table 2 and 3, and using theme complex, main theme, subtheme, and exemplary quotations for every theme. Diverse cases, e.g. a range of different opinions, views and preferences of the participants were represented in the quotes. Differences found between groups were reported in the results section and summarized at the end of the results section in “***Similar and Distinctive Themes Between Groups of Participants”***. Minor concepts were addressed in the discussion and referred to the range of patients’ experience. |
| Clarity of minor themes | 32 | Is there a description of diverse cases or discussion of minor themes? |  |
